# Frailty and Elder Abuse Among Older Adults in Outpatient Settings in Nigeria: A Case-Control Study

**DOI:** 10.1192/j.eurpsy.2026.10672

**Published:** 2026-07-31

**Authors:** E. L. Allagoa, C. G. Piwuna, U. Mariere, S. Reuben

**Affiliations:** 1 Department of Psychiatry, Lancaster University, Lancaster, United Kingdom; 2 Department of Public Health, Federal Medical Centre Yenagoa, Yenagoa; 3 Department of Psychiatry, Jos University Teaching Hospital Jos, Jos, Nigeria; 4 Adult CMHT Preston, Lancashire and South Cumbria NHS Foundation Trust, Preston, United Kingdom; 5 Department of pathology, St George University, True Blue, Grenada

## Abstract

**Introduction:**

Background

Frailty and elder abuse are growing concerns among older adults, especially in low-resource settings like Nigeria. Frailty may increase vulnerability to abuse, yet evidence from outpatient psychiatric and general medical settings remains limited (Dong, 2015; Yon et al., 2017; Torres-Castro et al., 2018).

**Objectives:**

To assess and compare the prevalence of elder abuse and frailty among older adults in General Outpatient (GOPD) and Psychiatric Clinics in a Nigerian teaching hospital, Jos University teaching Hospital, Jos and to explore associations with sociodemographic factors.

**Methods:**

A case-control study of 140 adults aged ≥50 years (75 GOPD, 65 Psychiatry). Elder abuse, frailty, and sociodemographic data were collected using structured questionnaires. Analyses included chi-square and Mann-Whitney U tests (Fulmer et al., 2005; Cooper et al., 2009; Bouillon et al., 2013), with significance at p < 0.05.

**Results:**

Psychiatric patients were significantly older (p = 0.015) and more often female (p = 0.005). Overall elder abuse prevalence was high (69.3%), significantly greater in psychiatric outpatients (78.5%) than GOPD (61.3%; p = 0.028). Psychological, neglect, and financial abuse were particularly elevated in psychiatric patients (Dong et al., 2013; Santos et al., 2019). Frailty severity showed no significant difference between groups, and no association with abuse was identified (Shamliyan et al., 2013; Torres-Castro et al., 2018).

**Table 1.** Overral Elder Abuse Prevalence Table 1. Long description.

Group Elder	Abuse %	No abuse %
GOPD	61.3	38.7
Psychiatry	78.5	21.5

**Table 2.** Types of elderly abuse Table 2. Long description.

Type of abuse	GOPD %	Psychiatry %
Psychological	50.7	69.2
Neglect	14.7	46.2
Financial	33.3	55.4
Physical	21.3	33.8
sexual	6.7	13.8

**Table 3.** Table 3. Long description.

Median Frailty	Score
GOPD	2.0
Psychiatry	2.0

**Conclusions:**

Elder abuse is highly prevalent in Nigerian outpatient settings, particularly among psychiatric patients. Frailty was not independently associated with abuse, but their co-occurrence highlights the need for integrated screening and culturally adapted interventions in routine care (Yon et al., 2017; Bouillon et al., 2013).

**Keywords:** Elder abuse; Frailty; Psychiatry; General Outpatients; Jos; Nigeria

**Disclosure of Interest:**

None Declared

## Long descriptions

### Table 1. Long description

The table consists of three columns: Group Elder, Abuse %, and No abuse %.

* The first data row for G O P D shows 61.3% for Abuse and 38.7% for No abuse.

* The second data row for Psychiatry shows 78.5% for Abuse and 21.5% for No abuse.

Navigate back to Table 1..

### Table 2. Long description

The table consists of three columns: Type of abuse, G O P D percentage, and Psychiatry percentage. The data is organized as follows:

* Psychological abuse: 50.7 percent in G O P D and 69.2 percent in Psychiatry.

* Neglect: 14.7 percent in G O P D and 46.2 percent in Psychiatry.

* Financial abuse: 33.3 percent in G O P D and 55.4 percent in Psychiatry.

* Physical abuse: 21.3 percent in G O P D and 33.8 percent in Psychiatry.

* Sexual abuse: 6.7 percent in G O P D and 13.8 percent in Psychiatry.

Across all categories, the Psychiatry department reported higher percentages of abuse than the G O P D.

Navigate back to Table 2..

### Table 3. Long description

The table consists of two columns and three rows including the header.

* The header row contains the labels Median Frailty in the first column and Score in the second column.

* The first data row lists G O P D in the first column with a score of 2.0 in the second column.

* The second data row lists Psychiatry in the first column with a score of 2.0 in the second column.

Navigate back to Table 3..

